# Impact of continuous glucose monitoring on quality of life, treatment satisfaction, and use of medical care resources: analyses from the SWITCH study

**DOI:** 10.1007/s00592-014-0598-7

**Published:** 2014-07-19

**Authors:** E. Hommel, B. Olsen, T. Battelino, I. Conget, I. Schütz-Fuhrmann, R. Hoogma, U. Schierloh, N. Sulli, H. Gough, J. Castañeda, S. de Portu, J. Bolinder, The SWITCH Study Group

**Affiliations:** 1 Steno Diabetes Center, Niels Steensensvej 2, 2820 Gentofte, Copenhagen, Denmark; 2 Herlev University Hospital, Copenhagen, Denmark; 3 University Children’s Hospital, University of Ljubljana, Ljubljana, Slovenia; 4 Diabetes Unit, ICMDM Hospital Clínic i Universitari, Barcelona, Spain; 5 Hospital Hietzing, Vienna, Austria; 6 Groene Hart Ziekenhuis, Gouda, The Netherlands; 7 Centre Hospitalier de Luxembourg, Clinique Pediatrique, Luxembourg, Luxembourg; 8 Clinica Pediatrica, Servizio Diabetologia, Policlinico Umberto I, Rome, Italy; 9 Medtronic International Trading Sarl, Tolochenaz, Switzerland; 10 Medtronic Bakken Research Centre, Maastricht, The Netherlands; 11 Department of Medicine, Karolinska Institutet, Karolinska University Hospital Huddinge, Stockholm, Sweden

**Keywords:** Continuous glucose monitoring, Medical resources, Insulin pump therapy, Quality of life, Sensor-augmented insulin pump therapy, Treatment satisfaction

## Abstract

To investigate the impact of continuous glucose monitoring (CGM) on health-related quality of life (HRQOL), treatment satisfaction (TS) medical resource use, and indirect costs in the SWITCH study. SWITCH was a multicentre, randomized, crossover study. Patients with type 1 diabetes (*n* = 153) using continuous subcutaneous insulin infusion (CSII) were randomized to a 12 month sensor-On/Off or sensor-Off/On sequence (6 months each treatment), with a 4-month washout between periods. HRQOL in children and TS in adults were measured using validated questionnaires. Medical resource utilization data were collected. In adults, TS was significantly higher in the sensor-On arm, and there were significant improvements in ratings for treatment convenience and flexibility. There were no clinically significant differences in children’s HRQOL or parents’ proxy ratings. The incidence of severe hypoglycaemia, unscheduled visits, or diabetes-related hospitalizations did not differ significantly between the two arms. Adult patients made fewer telephone consultations during the sensor-On arm; children’s caregivers made similar numbers of telephone consultations during both arms, and calls were on average only 3 min longer during the sensor-On arm. Regarding indirect costs, children with >70 % sensor usage missed fewer school days, compared with the sensor-Off arm (*P* = 0.0046) but there was no significant difference in the adults days of work off. The addition of CGM to CSII resulted in better metabolic control without imposing an additional burden on the patient or increased medical resource use, and offered the potential for cost offsets.

## Introduction

The diabetes control and complications trial (DCCT) has clearly shown that intensive glycaemic control reduces the risk of microvascular and macrovascular complications in patients with type 1 diabetes mellitus [1, 2], but such control is often difficult to achieve [2–4]. In recent years, intensive glycaemic control has been facilitated by technological developments such as continuous subcutaneous insulin infusion (CSII) [5] and continuous glucose monitoring (CGM) [6].

When attempting to establish optimal glycaemic control, different challenges arise depending on the age of the patient. Glycaemic control may be particularly difficult to achieve in children, because of the unpredictability of blood glucose levels, even with pump therapy, and patient or caregiver concerns about hypoglycaemia [7]. CSII is a safe and effective therapy providing physiological insulin delivery and features that assist improved diabetes management [8, 9]. In children, the risk of serious adverse events is similar between MDI and CSII [10]. The rate of discontinuation of pump therapy in children and adolescents has been associated with failure to improve HbA1c [8]. The recent SWITCH (Sensing with Insulin pump Therapy to Control HbA1c) study investigated the impact on metabolic control of combining CGM with CSII therapy in a mixed population of paediatric and adult patients with type 1 diabetes [11]. This study showed that the combination of CGM with CSII resulted in significant reductions in mean HbA1c levels and in the proportion of time with hypoglycaemia in both children and adults, compared with CSII alone. After 6 months, the mean decrease in HbA_1c_ during CGM was −0.4 %, 95 % CI −0.3, −0.6 %, (−5 mmol/mol, 95 % CI −4, −6 mmol/mol) (*P* < 0.001). The mean decreases in adults and children were −0.4 %, 95 % CI −0.3, −0.5 % (−4 mmol/mol, 95 % CI −3, −6 mmol/mol) and −0.5 %, 95 % CI −0.3, −0.7 % (−5 mmol/mol, 95 % CI −3, −7 mmol/mol), respectively. In addition, the total number of boluses per day was significantly larger (6.8 ± 2.5 vs 5.8 ± 1.9, *P* < 0.0001) during the sensor-On arm, compared with the sensor-Off arm, but the mean total daily insulin dose did not differ significantly between the two arms, indicating a change in therapy management by the subjects. There was no significant difference in the incidence of severe hypoglycaemic events or diabetic ketoacidosis events between the two arms [11].

In the current environment, improving outcomes and demonstrating value are crucial for the introduction of new technologies such as CGM into clinical practice. For this reason, we assessed the effects of CGM on health-related quality of life (HRQOL), treatment satisfaction, medical resource utilization, and time lost from school or work. The cross-over population of the SWITCH study gave a unique opportunity to perform this analysis using validated age-specific questionnaires.

## Methods

Details of the SWITCH study have been presented in full elsewhere [12]. In brief, the study was a randomized, controlled, crossover study involving patients aged between 6 and 70 years with type 1 diabetes of more than 1 year and an HbA_1c_ level between 7.5 and 9.5 % (59–80 mmol/mol). All patients had been using CSII with rapid-acting insulin analogues for at least 6 months, but had not previously used CGM. In addition, all patients had successfully completed a five-question multiple-choice test concerning pump therapy and general understanding of diabetes. Following a 1-month run-in period, patients were randomized to CGM sensor-On/sensor-Off or sensor-Off/sensor-On treatment sequences for 6 months each, with a 4-month washout phase between the two periods to minimize potential carryover effects. All patients used an insulin pump system (Mini-Med Paradigm REAL-Time System and Medtronic SofSensor; Medtronic, Tolochenaz, Switzerland) throughout the study. Data were uploaded using diabetes management software (CareLink Therapy Management System for Diabetes—Clinical; Medtronic, Tolochenaz, Switzerland) at scheduled study visits which took place at 6-week intervals during each treatment period. Patients used a continuous glucose monitor (Guardian REAL-Time Clinical; Medtronic, Tolochenaz, Switzerland), to which they were blinded (the device screen was switched off) for 2 weeks prior to randomization, prior to the second study period (i.e. during the crossover period), and during the sensor-Off arm. No fixed treatment algorithms were provided to the participants. The centre variable was significant in the ANOVA model (*P* < 0.0001); however, the interaction of centre and treatment was not (*P* = 0.9306) [11].

### Measurement of HRQOL and treatment satisfaction

HRQOL in children and adolescents (age ≤18 years) was measured by means of the Paediatric Quality of Life Inventory (PedsQL™), version 4.0 [13], which was completed by both children and their parents. This consists of age-specific (6–12 years; 12–18 years) questionnaires, with 23 questions covering four domains (physical, emotional, social functioning, and school functioning); higher scores indicate better HRQOL, and changes in PedsQL scores of between 4.36 and 9.67 points, depending on the domain, are considered clinically significant [14]. Treatment satisfaction in adults was measured by means of the Diabetes Treatment Satisfaction Questionnaire, status version (DTSQs) [15, 16]. The DTSQs consists of eight items (score 0–6) assessing current therapy, convenience, flexibility, diabetes knowledge, willingness to continue with therapy, willingness to advocate treatment, perceived hypoglycaemia, and perceived hyperglycaemia. The validated treatment satisfaction questionnaire was only available for adults, hence could not be conducted in the paediatric population. Due to the multicentre nature of the study a satisfactory validated adult, diabetes-specific QOL questionnaire was not available in all necessary languages.

### Use of medical resources

Data on medical resource use (diabetes-related hospitalizations and their duration, diabetes-related emergency department visits, unscheduled visits, and number and length of telephone consultations) and time lost from school or work were collected on the electronic case report form at each study visit in both arms.

### Statistical methods

All analyses were performed on the intention-to-treat (ITT) population, which included all randomized patients irrespective of their adherence to treatment or protocol violations. The per-protocol population (PP) included patients compliant with the protocol who used the sensor at least 70 % of the required time. Missing data within each treatment period were replaced according to the last observation carried forward (LOCF) principle, and possible carryover effects were minimized using a 4-month washout period [12]. HbA_1c_ levels in the sensor-On and sensor-Off treatment periods were compared by means of an analysis of covariance (ANCOVA) model with adjustment for period effect and subject as random effect. The average daily number of finger-prick tests for self-monitoring of blood glucose (SMBG), and the average daily insulin dose were compared in the two treatment arms using a mixed model with subject as random effect and adjustment for period effect and age group (children/adolescents or adults).

The analysis of factors predictive of end-of-period HbA_1c_ was performed using a multiple imputation linear mixed model adjusted for period effects. Factors including sequence, treatment, age group, baseline HbA_1c_, average total daily dose, SMBG, hospitalizations, telephone calls, and additional visits, number of boluses, basal rates, and bolus wizard use were considered.

Treatment satisfaction in adults was analysed by linear mixed models. DTSQs perceived frequency of hyperglycaemia and perceived frequency of hypoglycaemia were treated individually in these analyses, as per DTSQs user instructions. HRQOL in children and adolescents was analysed using linear mixed models adjusted for baseline HbA_1c_, study period, age group (5–7, 8–12, 13–17 years), and percentage of sensor usage. Generalized linear mixed models were used to analyse the occurrence of hospitalizations and additional health care visits, the duration of hospitalizations, the number of additional visits, and the number of telephone consultations during each treatment period.

Analyses were performed using SAS (version 9.3) software (SAS Institute, Cary, NC, USA), and *P* values below 0.05 were considered significant.

## Results

A total of 153 patients with type 1 diabetes (81 adults, 72 children and adolescents) were randomized, 77 to the On/Off sequence and 76 to the Off/On sequence. Overall, 15 patients withdrew during the study, but all randomized patients were included in the analysis of the primary endpoint, the difference in HbA_1c_ between the sensor-On and sensor-Off arms. All 72 children and 79 adults were included in analyses of patient-reported outcomes.

### HRQOL and treatment satisfaction

Changes in children’s self-reporting of HRQOL, and parents’ proxy assessments, between the Sensor-On and Sensor-Off arms are summarized in Table 1. There were no significant changes in children’s self-reports in overall HRQOL scores or in any HRQOL domain, as assessed by the PedsQL. There were statistically significant decreases in parents’ proxy ratings for the total PedsQL score and for all domains except the emotional domain, but in each case, the changes were smaller than those considered being clinically significant [14], and hence they were not regarded as clinically relevant.

**Table 1:** Changes in children’s self-reported health-related quality of life (HRQOL) and parents’ proxy ratings, between the sensor-On and sensor-Off arms

PedsQL™ domain	Child’s self-rating (*n* = 72)		Parents’ proxy rating (*n* = 72)	
	Mean (±SE) change	*P*	Mean (±SE) change	*P*
Physical	−0.11 ± 1.01	0.917	−4.22 ± 1.35	0.003^a^
Psychosocial	−0.59 ± 1.19	0.623	−5.08 ± 1.59	0.002^a^
Emotional	0.53 ± 1.54	0.734	−1.57 ± 1.57	0.318^a^
Social	−0.35 ± 0.94	0.715	−3.75 ± 1.51	0.015^a^
School	−1.40 ± 1.63	0.396	−6.14 ± 1.84	0.001^a^
Overall HRQOL	−0.31 ± 0.84	0.712	−3.92 ± 1.18	0.002^a^

HRQOL was measured using the paediatric quality of life inventory (PedsQL™) [10]

^a^Clinical relevant changes were as defined by Jaeschke et al. [14]

In adults, treatment satisfaction measured by the DTSQs increased significantly during the sensor-On arm, compared with the sensor-Off arm (*P* = 0.012, Table 2), and this was associated with significant improvements in treatment convenience (*P* = 0.033) and flexibility (*P* = 0.034). There were no significant differences in the perceived frequency of hypoglycaemia or hyperglycaemia during the sensor-On arm. A per-protocol analysis showed that sensor use was significantly (*P* = 0.027) associated with treatment satisfaction.

**Table 2:** Change in treatment satisfaction measured using the Diabetes Treatment Satisfaction Questionnaire status version (DTSQs) [12] in the sensor-On arm, compared with the sensor-Off arm

DTSQs item	Change versus baseline	*P*
Current therapy	0.19	0.062
Perceived hyperglycaemia	−0.23	0.231
Perceived hypoglycaemia	0.22	0.205
Convenience	0.32	0.033
Flexibility	0.29	0.034
Diabetes knowledge	0.06	0.466
Continue with therapy	0.20	0.075
Advocate treatment	0.15	0.15
Overall treatment satisfaction	1.16	0.010

### Medical resource use

Use of medical care resources during the sensor-On and sensor-Off arms is summarized in Table 3, and an analysis by age group for those items where significant differences between arms were observed is summarized in Table 4. The number of telephone calls showed significant difference between arms (Table 3). Analysis by age group showed that adult patients made significantly fewer additional telephone consultations during the sensor-On period, compared with the sensor-Off period (Table 4). In children, during the sensor-On period, telephone consultations were approximately 3 min longer on average (*P* = 0.0055), compared with the sensor-Off arm. In both adults and children, there were no statistically significant differences in the number of diabetes-related hospitalizations between the sensor-On and sensor-Off arms, although the mean duration of hospitalization tended to be shorter during the sensor-On arm (1.80 vs 2.33 days, respectively).

**Table 3:** Mean (±SD) use of health care resources during the sensor-On and sensor-Off arms for the total population

	Sensor-On	Sensor-Off	Difference	*P*
Total daily insulin dose (U100, units/day)	48.9 ± 3.7	47.3 ± 3.7	1.64	0.0638
Finger-prick tests per day	5.0 ± 0.3	5.5 ± 3.7	−0.56	<0.0001
At least one adverse event	44.9 % (36.4–53.7)^*^	50.4 % (41.7–59.1 %)^*^	−5.5 %	0.3467
At least one hospitalization	6.9 % (3.7–12.4 %)^*^	4.4 % (1.9–9.6 %)^*^	2.5 %	0.3634
At least one diabetes-related hospitalization	2.5 % (0.9–6.8 %)^*^	0.6 % (0.1–4.5 %)^*^	1.9 %	0.2138
Hospitalization duration (days)	1.8 (1.0–3.2)^*^	2.3 (1.1–5.0)^*^	0.5	0.5783
At least one additional visit	12.9 % (8.2–19.7 %)^*^	11.9 % (7.4–18.6 %)^*^	1.0 %	0.7997
Number of additional visits	0.1 (0.1–0.2)^*^	0.1 (0.1–0.2)^*^	0	0.9859
Number of additional telephone calls**	On/Off: 0.5 (0.4–0.8)^*^	On/Off: 0.4 (0.3–0.7^*^	On/Off: 0.1	On/Off: 0.3553
	Off/On: 0.5 (0.3–0.8)^*^	Off/On: 1.0 (0.7–1.4)^*^	Off/On: −0.4	Off/On: < 0.0001
Duration of additional telephone calls (min)	6.8 ± 1.0	5.5 ± 1.0	1.3	0.0696

^*^95 % CI ** results presented by treatment sequence

**Table 4:** Mean (±SD) number of finger-prick tests, number and duration of additional telephone calls, by age group (*NS* not significant)

	Children			Adults		
	Sensor-On	Sensor-Off	*P*	Sensor-On	Sensor-Off	*P*
Number finger-prick tests per day	4.6 ± 0.2	5.2 ± 0.2	<0.0001	5.4 ± 0.2	5.8 ± 0.2	0.0075
Number of additional telephone calls	On/Off: 0.8 ± 0.2	On/Off: 0.5 ± 0.1	0.0113	On/Off: 0.4 ± 0.1	On/Off: 0.4 ± 0.1	Ns
	Off/On: 0.7 ± 0.2	Off/On: 1.0 ± 0.2	Ns	Off/On: 0.3 ± 0.1	Off/On: 0.9 ± 0.2	<0.0001
Duration of additional telephone calls (min)	7.6 ± 0.6	4.7 ± 0.7	0.0055	6.0 ± 0.7	6.3 ± 0.7	Ns

In a per-protocol analysis, children who used their sensors more than 70 % of the time (i.e. ≥5 days per week) missed significantly less time from school during the sensor-On period, compared with the sensor-Off period (mean 0.38 vs 1.24 days/child/6 months, respectively, *P* = 0.0046). There was no significant difference in the per-protocol analysis of days lost from work in the adult cohort.

Of the factors modelled to predict end-of-period HbA1c, only the administration of one more bolus per day (*P* = 0.01) and the use of sensor (being on sensor-ON, *P* < 0.001) were associated with a decrease in HbA_1c_.

## Discussion

These results show that the addition of CGM in patients receiving CSII therapy improves metabolic control [11], reflected as lower HbA_1c_, can be accomplished without a significant burden on patients or an increase medical resource use. From the patient’s perspective, CGM does not adversely affect HRQOL in children, while adults report greater satisfaction with treatment. Indeed, this was confirmed by the per-protocol analysis showing positive association between sensor usage >70 % and treatment satisfaction. This latter finding is important because treatment satisfaction has been suggested to be an indicator of better outcomes in chronic conditions such as diabetes [16]. From the perspective of the healthcare system, there was no difference in the number of visits or of diabetes-related hospitalizations, although the duration of hospitalization tended to be shorter during the Sensor-On arm. There was also no increased exposure to severe hypoglycaemia, and the time spent with blood glucose below 70 mg/dl was significantly reduced [11]. The incidence of diabetic ketoacidosis episodes was very low and did not differ between the two arms.

It could be anticipated that the introduction of CGM may increase anxiety and burden for both parents and patients, resulting in a decrease in their HR-QOL. Although the results were not significant in children, there was a tendency towards negative results in all domains except emotional. For the parents, this negative impact could have resulted from a too short follow-up period to ameliorate this anxiety. Surprisingly, the lack of burden or deterioration in QOL in children could show either a faster adaptation to the therapy or that the value of the device outweighed the burden.

The SWITCH study has shown that increasing the number of boluses [11] and wearing the sensor (sensor-On) are predictive of reducing end-of-period HbA_1c_.

Each centre in the study decided how to train subjects in the study, whether individually or in groups. Three study visits were allocated to education, which could take up to 1 h each. This may have resulted in the high sensor usage reported in the study [11] as education could enhance therapy motivation and may also explain the higher TS in the per-protocol population. Adult patients made more telephone consultations in the sensor-Off arm. The children made longer telephone consultations during the sensor-On arm; these calls were only 3 min longer. This suggests that the sensor assists self-management in adults, whereas the additional information provided by CGM in children may require an additional 3 min of healthcare professional time during the initiation of CGM, to advise and train effectively.

Children who used the sensor frequently missed significantly fewer days of school, which could in turn result in parents missing fewer days of work. Parents missing work to care for children were not measured as part of the adult cohort missed days work, which was not significant. Thus, the use of CGM could offer potential savings for society. Together, these results suggest that the addition of CGM to CSII results in better metabolic control without imposing an additional burden on the patient or increasing medical resource use and offers the potential for cost offsets.

These findings are consistent with those of a previous study from the Juvenile Diabetes Research Foundation (JDRF) CGM trial, which found little change in QOL measures in children and adults using CGM, compared with patients using self-monitoring of blood glucose, although patients reported high levels of satisfaction with CGM [17]. Similarly, in the STAR (Sensor-augmented pump Therapy for A1c Reduction)-3 study, the use of CGM with pump therapy was found to offer significant advantages in terms of treatment satisfaction for both adult and paediatric patients and their caregivers [18]. These high levels of treatment satisfaction are related both to factors such as convenience and flexibility, as in the present study, and to decreased concerns about hypoglycaemia [7, 17–19]. The SWITCH study results are also consistent with observational studies in everyday life that show that the use of CGM resulted in an even greater decrease in the number of SMBG performed. [19, 20].

There is evidence that CGM has the potential to offer both short-term and long-term benefits, not only in terms of clinical outcomes, but also in HRQOL in patients with type 1 diabetes. Short-term benefits may arise as a result of both easier decision-making about insulin therapy and decreased fear of hypoglycaemia, whereas long-term benefits may be attributable to the avoidance of vascular complications resulting from intensive glycaemic control [21]. The SWITCH showed that adding CGM improved short-term treatment satisfaction in adults and that the introduction of a new technology does not have a significant negative impact on children’s quality of life.

The results may have been improved using the DTSQ change version (DTSQc), which is more responsive to improvements in treatment satisfaction than the DTSQs, especially when there are ceiling effects [22]. Thus, the DTSQs may be less sensitive to changes in patients who are already satisfied with their treatment. However, it is noteworthy that no decline in treatment satisfaction was seen in the Sensor-On arm and that there were significant improvements in ratings for treatment convenience and flexibility.

In terms of study limitations, it should be noted that all patients were experienced users of modern diabetes technology (all patients were required to have been using CSII for at least 6 months, although they had not previously used CGM), so these findings may not apply to all patients with type 1 diabetes. Instruction in the use of CGM requires clinician–patient contact, and there are currently no agreed protocols for the incorporation of CGM into routine clinical practice [23]. Another limitation is that the duration of additional telephone consultations was self-reported by study staff on the electronic case report form. In addition, treatment satisfaction was measured only in adults as no validated paediatric TS questionnaire was available; hence, HRQOL was measured only in children.

In conclusion, the addition of CGM to CSII improves metabolic control concomitantly reducing the time spent in hypoglycaemia. Treatment satisfaction is increased in adults, and there is no adverse effect on self-reported quality of life in children. Importantly, these benefits are achieved without significantly increasing medical resource use. From the health care perspective, both the potential long-term benefit of improved metabolic control in terms of reducing long-term complications [1, 2] and the potential for short-term benefits may offer opportunities for cost offsets. The SWITCH study adds to the growing body of evidence for the convincing benefits of CGM in terms of health outcomes, HRQOL, treatment satisfaction, and medical resource use.
